# Tertiary Epimutations – A Novel Aspect of Epigenetic Transgenerational Inheritance Promoting Genome Instability

**DOI:** 10.1371/journal.pone.0168038

**Published:** 2016-12-19

**Authors:** John R. McCarrey, Jake D. Lehle, Seetha S. Raju, Yufeng Wang, Eric E. Nilsson, Michael K. Skinner

**Affiliations:** 1 Department of Biology, University of Texas at San Antonio, San Antonio, TX United States of America; 2 Center for Reproductive Biology, School of Biological Sciences, Washington State University, Pullman, WA United States of America; Massachusetts General Hospital, UNITED STATES

## Abstract

Exposure to environmental factors can induce the epigenetic transgenerational inheritance of disease. Alterations to the epigenome termed “epimutations” include “primary epimutations” which are epigenetic alterations in the absence of genetic change and “secondary epimutations” which form following an initial genetic change. To determine if secondary epimutations contribute to transgenerational transmission of disease following in utero exposure to the endocrine disruptor vinclozolin, we exposed pregnant female rats carrying the *lacI* mutation-reporter transgene to vinclozolin and assessed the frequency of mutations in kidney tissue and sperm recovered from F1 and F3 generation progeny. Our results confirm that vinclozolin induces primary epimutations rather than secondary epimutations, but also suggest that some primary epimutations can predispose a subsequent accelerated accumulation of genetic mutations in F3 generation descendants that have the potential to contribute to transgenerational phenotypes. We therefore propose the existence of “tertiary epimutations” which are initial primary epimutations that promote genome instability leading to an accelerated accumulation of genetic mutations.

## Introduction

Since the initial report of the induction of epigenetic transgenerational inheritance of adult-onset disease in F3 generation progeny from an F0 generation gestating female rat exposed to the endocrine disruptor vinclozolin [1], there have been numerous reports of similar effects caused by exposure of fetuses or adults to a variety of different environmental factors (see [2,3] for reviews), as well as reports of epigenetic transgenerational inheritance in numerous additional species [1,4–9]. These environmental factors include various toxicants, including many different endocrine disruptors, nutrition effects including famine, caloric restriction, high fat diets or folate deficiencies, and other stressors such as drought, smoking, alcohol or heat. Exposure of fetuses to these agents in utero, or of adults to these environmental factors, is associated with transgenerational increases in the incidence of adult-onset disease or abnormalities impacting the reproductive systems and fertility in both sexes, the incidence of cancer, the immune system, kidney function, the prostate, obesity, cardiovascular function, growth, insulin sensitivity, glucose tolerance, pulmonary function, neuronal function and social behavior, among other effects [3]. These reports have revealed a novel etiology of adult-onset disease and a mechanism underlying the developmental origins of health and disease (DOHAD) [10]. In addition, these observations suggest that the phenomenon of epigenetic transgenerational inheritance of disease is significantly more extensive than previously thought.

While it is not surprising that exposure to environmental factors or toxicants can alter the epigenome, especially during key developmental periods when epigenetic reprogramming is taking place [11], it is surprising that such alterations, known as epimutations, can persist over multiple generations in the absence of any further exposure to the disruptive agent [12–14]. Persistence of abnormalities for one or two generations following in utero exposure may simply reflect toxicity related effects induced by direct exposure of an F0 generation female, an F1 generation fetus, and/or the germ cells within that fetus that will give rise to the F2 generation. This phenomenon has been termed a “multigenerational exposure” [15] or more recently “intergenerational inheritance” [16], but, in fact, does not really represent true “inheritance” at all if it is limited to direct exposure toxicity effects. However, transmission of phenotypes through three or more generations following a single transient exposure to the causative agent requires some mechanism of transgenerational inheritance [1,13,17]. Epimutations induced by certain effects, for example the use of assisted reproductive technologies (ART), have been shown to be corrected by normal germline-specific epigenetic reprogramming such that they are detected in the F1 generation, but are not transmitted to subsequent generations [18]. These are examples of intergenerational epimutations that are not transgenerational. This begs the question of how epimutations caused by exposure to certain environmental factors, such as endocrine disruptors, during critical windows of development are able to avoid subsequent correction by germline epigenetic reprogramming such that they persist over multiple generations via transgenerational inheritance.

One potential explanation for the observed transgenerational transmission of epimutations induced by a single exposure to vinclozolin or similar agents in utero is that this exposure could promote the formation of genetic mutations that impact epigenetic programming, such that the phenotype of these mutations is manifest as epimutations, but the underlying source of these defects is one or more genetic mutations. Indeed, two distinct types of epimutations have been previously described [19]. “Primary epimutations” are epigenetic aberrations that occur in the absence of any genetic change and are propagated via mitosis to daughter cells or via meiosis to subsequent generations on the basis of epigenetic rather than genetic inheritance. “Secondary epimutations” are those that form as a consequence of, and thus subsequent to, an initial genetic change (mutation), and can therefore be transmitted on the basis of genetic transmission of the initial mutation or on the basis of epigenetic transmission of the subsequent epimutation, or both. Primary epimutations in the germ line would have the potential to be corrected by germline epigenetic reprogramming, whereas secondary epimutations would not be corrected so would be transmitted transgenerationally as genetic traits. Thus to understand the mechanism of epigenetic transgenerational inheritance, it is important to distinguish between primary and secondary epimutations.

Mutation-reporter transgenes provide a convenient and sensitive approach to assess the frequency of genetic mutations. Such systems can facilitate testing of tens or hundreds of thousands of copies of a reporter transgene to accurately determine the frequency of point mutations in this gene without the need to sequence most of these copies. One of the most extensively studied mutation-reporter systems is the “Big Blue” rodent system originally developed by Stratagene (now part of Agilent) in the 1990s [20,21]. This approach provides a method to examine the frequency of mutations accumulated in a transgene that is integrated into the same genomic location in all cells, but is not expressed in any cell type of the animal so does not impact the function of those cells in any way. Specifically, the Big Blue system is based on the colorimetric detection of expression of the *lacZ* reporter gene as an indicator of mutations that have occurred in the *lacI* (repressor) gene. Copies of the transgene expressing the mutant phenotype are sequenced to confirm the presence and type of the relevant mutation in the *lacI* gene and to identify clonal mutations which are counted as a single mutagenic event regardless of how many times they are detected in the same sample.

To determine if in utero exposure to vinclozolin leads to the direct induction of genetic mutations, and, thus, potentially to the induction of secondary epimutations, we exposed pregnant female Big Blue Rats carrying the *lacI* mutation-reporter transgene to vinclozolin as previously described [1,10] and compared the frequency of mutations in the *lacI* mutation-reporter transgene in kidney tissue and sperm recovered from F1 generation offspring of vinclozolin-treated or control (vehicle-treated) dams. In addition, we bred F1 generation offspring to generate both vinclozolin- and control-lineage F2 generation and subsequently F3 generation descendants as described [3,22], and analyzed the frequency of spontaneous mutations in kidney and sperm cells from the F3 generation progeny as well. Our results reveal the unexpected finding of a third type of epimutation which we term “tertiary epimutations.” Tertiary epimutations are distinct from primary or secondary epimutations and have the potential to contribute to epigenetic transgenerational inheritance.

## Materials and Methods

### Samples

The *lacI* mutation-reporter transgene (‘Big Blue’) system has been used extensively for the assessment of relative mutation frequencies in different animals, cell types and/or samples for nearly 25 years [23,24]. Big Blue rats homozygous for 2–4 copies of the *lacI* mutation-reporter transgene per genome [25] were obtained from Stratagene (La Jolla, CA). In the Skinner lab the Big Blue rats at 70 to 100 days of age were fed ad lib with a standard rat diet and ad lib tap water. To obtain timed-pregnant females, female rats in proestrus were pair-mated with male rats. Sperm-positive (day 0) rats were monitored for diestrus and body weight. On days 8–14 of gestation, the females were administered daily intraperitoneal injections of vinclozolin (100 mg/kg BW/day) or dimethyl sulfoxide (vehicle) as a control as described [26]. Generally sibling females were used for the control and vinclozolin lineage F0 generation females, respectively. In addition, sibling F0 generation males were used to generate control and exposure lineage offspring. A minimum of three distinct lineages were used for each exposure. Vinclozolin was obtained from Chem Service Inc. (West Chester, PA), and was injected in a 200 microliter DMSO/sesame oil vehicle as previously described [27].

The initial gestating female rats and the males to which they were mated were designated as the F0 generation. Offspring of F0 generation matings formed the F1 generation. Non-sibling females and males aged 70–90 days from F1 generation control or vinclozolin lineages were bred to generate F2 generation offspring. F2 generation rats were then bred to obtain F3 generation offspring. Only the F0 generation gestating female was directly treated transiently with vinclozolin. Different F0 generation females were used for each different experiment and one male per litter was selected as the individual animal to be analyzed for each specific experiment. Control and vinclozolin lineage animals were housed in the same room and racks with identical lighting and food + water supply as previously described [10,27,28]. Samples for analysis were recovered from F1 and F3 generation vinclozolin-lineage and control-lineage male descendants to distinguish between potential immediate mutagenic effects (F1 generation samples) and an indirect, subsequent increase in mutation frequencies (F3 generation samples). To collect tissue or cell samples, rats were placed into a chamber and exposed to CO2 until breathing ceased, followed by cervical dislocation as a secondary euthanasia method.

Kidney and sperm were selected as the cell types to be analyzed because pathological defects have previously been documented in each of these cell types recovered from both F1 and F3 generation male descendants of dams exposed to vinclozolin [1–3,10]. In addition, this provided one example of a somatic tissue (kidney) and one example of germline cells (sperm) among the cell types to be analyzed for mutation frequencies. To obtain sperm samples, the epididymis was dissected free of connective tissue and a small cut was made in the cauda portion, which was then placed in 5 ml of F12 culture medium containing 0.1% bovine serum albumin for 10 minutes at 37°C to allow sperm to emerge from the tissue and then kept at 4°C to immobilize the sperm. The epididymal tissue was minced and the released sperm centrifuged at 13, 000 x *g* and stored in fresh nucleus isolation medium buffer at -70°C as described [10,27,28]. Kidneys were also dissected and snap frozen at -70°C. Each tissue or cell sample was shipped to the McCarrey lab on dry ice for further analysis (see below). All experimental protocols for the procedures with rats were pre-approved by the Washington State University Institutional Animal Care and Use Committee (IACUC approval # 02568–039), and carried out in accordance with relevant guidelines and regulations.

Epigenetic transgenerational phenotypes were not monitored in this study because the Big Blue rat model is maintained on the Fisher inbred rat genetic background which has previously been shown to repress the occurrence of such phenotypes [22], and because frequencies and spectra of mutations in the *lacI* reporter gene were assessed in animals at <1 year of age which is prior to the age at which transgenerational phenotypes typically become evident.

### Analysis of mutation frequencies and spectra

High molecular weight DNA was prepared from each tissue or cell sample using the RecoverEase kit protocol from Stratagene with modifications as described [29,30,31]. Briefly, each sample was homogenized with a Dounce tissue homogenizer, filtered through a 100 μm filter (Millipore, Billerica, MA), subjected to digestion with ribonuclease (RNace-it, Stratagene) and proteinase K, then drop dialyzed against 1X TE using a 0.025 μm dialysis membrane (Millipore).

High molecular weight DNA recovered from each kidney or sperm sample was subjected to a phage packaging reaction using Transpack packaging extract (Stratagene) following the manufacturer’s protocol. Resulting infectious phage particles were plated onto a 25cm x 25cm lawn of SCS-8 E. coli cells to facilitate formation of phage plaques (up to 15,000 plaque forming units [pfu]/plate).

The *lacI* gene normally encodes a repressor of the *lac* operon that inhibits production of beta galactosidase by infected E. coli [32]. However, point mutations incurred in the *lacI* gene while it was present as a transgene in the cells of the Big Blue rats can result in defective function of the *lac* repressor such that it fails to repress expression of the *lac* operon, resulting in production of beta galactosidase by E. coli infected with phage carrying a mutated *lacI* gene. The addition of X-gal and IPTG to the plating medium facilitated a blue/clear color selection method to distinguish rare mutant phage expressing beta galactosidase (blue plaques) from abundant wild type phage in which expression of beta galactosidase remained repressed (clear plaques) on each plate. Plaques displaying an apparent mutant (blue) phenotype were then picked and re-plated to confirm the mutant phenotype in ≥ 50% of the resulting phage. Plaques carrying an abundance of mutant phage were then prepared for Sanger sequencing of the *lacI* gene to confirm the presence of a mutation at the molecular level and to determine the position and type of mutation present. Sequencing was outsourced to the DNA Sequencing Facility at UT Austin.

DNA sequence data from each mutant plaque was used to determine the final frequency of spontaneous point mutations in each sample. Only confirmed independent (non-clonal) mutations were used to calculate the final frequency of mutations. Thus, if the same mutation in the same position within the *lacI* gene was detected more than once in the same DNA sample, it was assumed that this represented a single original mutation that was subsequently clonally amplified in the tissue or cell type, and such ‘clonal’ mutations were counted as a single mutation no matter how many times they were detected in a particular sample. Mutation frequencies were calculated by dividing the number of distinct, confirmed, non-clonal mutant plaques by the total number of pfu examined from each DNA sample. In general, sufficient total numbers of pfu were examined to identify at least five separate mutant plaques from each sample to facilitate statistical comparisons of the data from different samples. The DNA sequence data were also used to establish a mutation spectrum for each sample by cataloguing the relative prevalence of different types of point mutations detected in each sample, including base substitutions–transitions or transversions, small insertions, small deletions, or multiple base changes.

Numbers of mutations were analyzed by a Poisson model with parameter estimates obtained by the method of maximum likelihood [33]. Because of the low expected frequencies, exact P-values were calculated by the exact conditional test for Poisson variables to compare differences between mutation frequencies, using the Exactci package implemented in R [34,35]. P ≤ 0.05 was considered statistically significant. Note that the extent of the standard error associated with each mutation frequency is dictated by the number of pfu examined and the number of independent mutations detected in those pfu.

## Results

### Mutation frequencies

**Fig 1:**
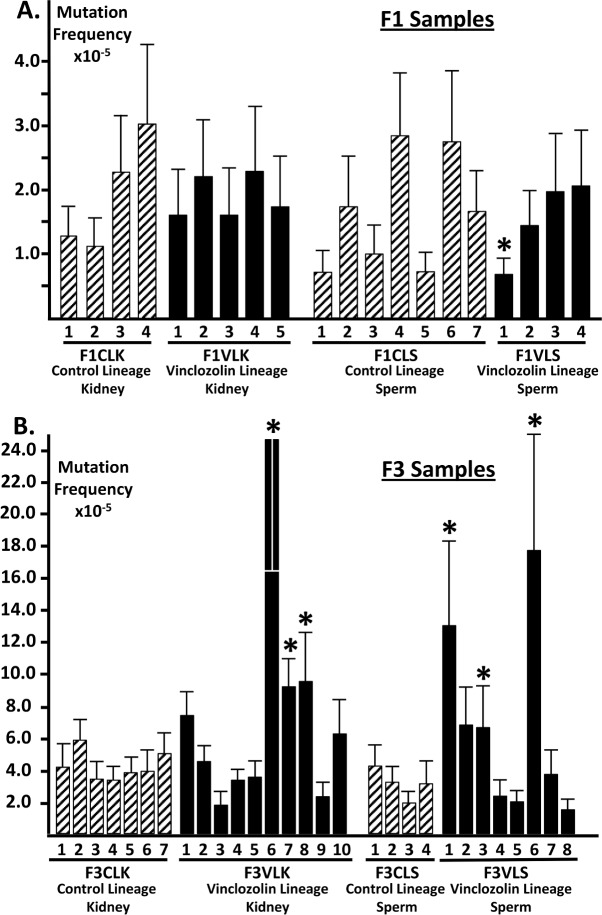
Mutation frequencies in F1 and F3 generation samples. **(A)** Mutation frequencies in kidney and sperm samples from F1 generation control- and vinclozolin-lineage animals. There were no statistically significant differences among the mutation frequencies detected in kidney or sperm samples from F1 generation control- and vinclozolin-lineage samples, except for one of the vinclozolin-lineage sperm samples (F1VL–marked with an asterisk) which showed a mutation frequency that was significantly lower than the mean of the F1 generation control-lineage samples (p = 0.00352). **(B)** Mutation frequencies in kidney and sperm samples from F3 generation control- and vinclozolin-lineage animals. A subset of both kidney and sperm samples from F3 vinclozolin-lineage descendants showed mutation frequencies that were not significantly different than the mean of the corresponding F3 generation control-lineage samples, although several of the F3 generation vinclozolin-lineage samples trended higher than the mean of the corresponding F3 generation control-lineage samples. However another subset of both kidney and sperm samples from F3 generation vinclozolin-lineage descendants showed mutation frequencies that were significantly higher than the mean of the corresponding F3 control-lineage samples. These mutation frequencies are marked with astrices, and include those found in the following samples: F3VLK6 (p = 0.00342), F3VLK7 (p = 0.00131), F3VLK8 (p = 0.0222), F3VLS1 (p = 0.00185), F3VLS2 (p = 0.03611)) and F3VLS6 (p = 0.00018). F1 = samples from F1 generation descendants, F3 = samples from F3 generation descendants, CL = samples from control-lineage descendants, VL = samples from vinclozolin-lineage descendants, K = kidney samples, S = sperm samples.

Frequencies of point mutations detected in kidney and sperm cells from F1 generation offspring of dams exposed during pregnancy to either vehicle (DMSO) plus vinclozolin (vinclozolin-lineage animals) or vehicle alone (control-lineage animals) are shown in Table 1. No significant differences in frequencies of mutations were observed between vinclozolin- and control-lineage kidney or sperm samples, respectively, except for one vinclozolin-lineage sperm sample (marked with an asterisk in Fig 1A) in which we detected a mutation frequency that was significantly lower than the mean frequency of the F1 generation control-lineage sperm samples (p = 0.0352). Previous studies in the mouse revealed enhanced maintenance of genetic integrity in germline cells relative to that in differentiated somatic cells [31,36]. This distinction was not as robust between rat germ cells (sperm) and somatic cells (kidney). These data confirm previous reports that vinclozolin is not directly mutagenic [37], and suggest that neither the immediate phenotypic abnormalities observed in vinclozolin-lineage F1 generation offspring nor the transmission of those defects to the F2 generation can be ascribed to the direct induction of genetic mutations.

**Table 1:** Mutation Frequencies in F1 Generation Control- and Vinclozolin-Lineage Samples.

				Mutation
Sample	Tissue	Total	Mutant	frequency
Number	Type	pfu	plaques	± SE (x 10^−5^)
Control-lineage				
F1CLK1	Kidney	6,15,930	8	1.30 ± 0.459
F1CLK2	Kidney	5,35,547	6	1.12 ± 0.457
F1CLK3	Kidney	2,98,750	7	2.34 ± 0.886
F1CLK4	Kidney	1,97,925	6	3.03 ± 1.240
F1CLS1	Sperm	7,01,848	5	0.71 ± 0.319
F1CLS2	Sperm	2,87,672	5	1.74 ± 0.777
F1CLS3	Sperm	5,09,007	5	0.98 ± 0.439
F1CLS4	Sperm	3,17,744	9	2.83 ± 0.944
F1CLS5	Sperm	8,38,565	6	0.72 ± 0.292
F1CLS6	Sperm	2,55,000	7	2.75 ± 1.040
F1CLS7	Sperm	3,61,750	6	1.66 ± 0.677
Vinclozolin-lineage				
F1VLK1	Kidney	3,71,736	6	1.61 ± 0.659
F1VLK2	Kidney	2,71,845	6	2.21 ± 0.901
F1VLK3	Kidney	3,10,250	5	1.61 ± 0.721
F1VLK4	Kidney	2,18,275	5	2.29 ± 1.020
F1VLK5	Kidney	2,88,000	5	1.74 ± 0.776
F1VLS1	Sperm	13,23,987	9	0.68 ± 0.227
F1VLS2	Sperm	4,86,858	7	1.44 ± 0.543
F1VLS3	Sperm	2,53,250	5	1.97 ± 0.883
F1VLS4	Sperm	2,91,250	6	2.06 ± 0.841

Frequencies of mutations detected in kidney and sperm cells from vinclozolin- or control-lineage F3 generation descendants of dams exposed during pregnancy to either vehicle plus vinclozolin or vehicle alone, respectively, are shown in Table 2. Interestingly, for both the control- and vinclozolin-lineage animals the frequencies of mutations detected in the F3 generation were consistently higher than those detected in F1 generation animals. The F1 and F3 generation samples were not generated or analyzed simultaneously, however, the control and vinclozolin-exposed samples within each generation were generated and analyzed simultaneously. Therefore, the relevant comparison in each case is between control and vinclozolin-exposed samples within the same generation.

**Table 2:** Mutation Frequencies in F3 Generation Control- and Vinclozolin-Lineage Samples.

				Mutation
Sample	Tissue	Total	Mutant	frequency
Number	Type	pfu	plaques	± SE (x 10^−5^)
Control-lineage				
F3CLK1	Kidney	1,92,021	8	4.17 ± 1.470
F3CLK2	Kidney	3,44,629	20	5.80 ± 1.300
F3CLK3	Kidney	2,80,976	10	3.56 ± 1.130
F3CLK4	Kidney	4,08,408	14	3.43 ± 0.916
F3CLK5	Kidney	3,42,510	13	3.80 ± 1.050
F3CLK6	Kidney	2,29,108	9	3.93 ± 1.310
F3CLK7	Kidney	2,98,833	15	5.02 ± 1.300
F3CLS1	Sperm	2,33,588	10	4.28 ± 1.350
F3CLS2	Sperm	3,37,211	11	3.26 ± 0.984
F3CLS3	Sperm	3,63,529	7	1.93 ± 0.728
F3CLS4	Sperm	1,58,172	5	3.16 ± 1.410
Vinclozolin-lineage				
F3VLK1	Kidney	1,35,475	9	6.64 ± 2.210
F3VLK2	Kidney	4,84,872	22	4.54 ± 0.967
F3VLK3	Kidney	2,64,136	5	1.89 ± 0.847
F3VLK4	Kidney	6,81,660	23	3.37 ± 0.704
F3VLK5	Kidney	3,75,254	13	3.46 ± 0.961
F3VLK6	Kidney	36,358	9	24.75 ± 8.250
F3VLK7	Kidney	2,61,008	23	8.81 ± 1.840
F3VLK8	Kidney	94,996	9	9.47 ± 3.160
F3VLK9	Kidney	2,60,167	6	2.31 ± 0.942
F3VLK10	Kidney	1,28,520	8	6.22 ± 2.200
F3VLS1	Sperm	53,656	7	13.05 ± 4.930
F3VLS2	Sperm	1,17,677	8	6.80 ± 2.400
F3VLS3	Sperm	91,889	6	6.53 ± 2.670
F3VLS4	Sperm	2,62,559	7	2.67 ± 1.010
F3VLS5	Sperm	3,92,605	8	2.04 ± 0.720
F3VLS6	Sperm	33,843	6	17.73 ± 7.240
F3VLS7	Sperm	1,62,386	6	3.70 ± 1.510
F3VLS8	Sperm	3,44,389	5	1.45 ± 0.649

When compared to mutation frequencies detected in kidney and sperm samples from F3 generation control-lineage animals, one subset of the kidney samples (7 of 10) and one subset of the sperm samples (5 of 8) from F3 generation vinclozolin-lineage animals showed mutation frequencies that were not statistically different from the control values, although the mutation frequencies detected in some of these F3 vinclozolin-lineage samples trended higher than those in the F3 control-lineage animals (Fig 1B). However, another subset of kidney samples (3 of 10) and sperm samples (3 of 8) from F3 generation vinclozolin-lineage animals showed mutation frequencies that were significantly higher than the mean frequency in the corresponding control-lineage samples (marked with astrices in Fig 1B; p < 0.05). Indeed, mutation frequencies detected in these divergent samples were 2–13 fold higher than the mean mutation frequency detected among the corresponding F3 generation control-lineage samples. This suggests that in utero exposure to vinclozolin predisposes a subsequent accumulation of mutations at an accelerated rate in some descendants. This effect appears to occur in a gradual and stochastic manner, such that it is not detectable in any vinclozolin-lineage F1 generation offspring but is manifest in a subset of vinclozolin-lineage F3 generation descendants.

### Mutation spectra

The *lacI* gene from each mutant (blue) plaque recovered from each cell or tissue sample was sequenced to confirm that a mutation had occurred in each case and to determine the range of types of mutations associated with each cell sample. A comprehensive list of the position and type of each mutation detected in the *lacI* reporter gene recovered from each set of tissues is shown in S1 Table, and a summary of the spectrum of mutations detected in each set of samples is shown in Table 3.

**Table 3:** Mutation Spectra Detected in Control- and Vinclozolin-Lineage Samples.

Generation/				
Tissue	TS^1^	TV^2^	I/D^3^	DBS^4^
**F1 Generation**				
Control-lineage				
Kidney	6/(40.00)	2/(13.33)	6/(40.00)	1/(6.67)
Sperm	10/(40.00)	6/(24.00)	7/(28.00)	2/(8.00)
Total	16/(40.00)	8/(20.00)	13/(32.50)	3/(7.50)
Vinclozolin-lineage				
Kidney	8/(66.67)	2/(16.67)	2/(16.67)	0/(0)
Sperm	8/(50.00)	2/(12.50)	5/(31.25)	1/(6.25)
Total	16/(57.14)	4/(14.29)	7/(25.00)	1/(3.57)
**F3 Generation**				
Control-lineage	17/(22.37)	11/(14.47)	46/(60.53)	2/(2.63)
Kidney	10/(26.32)	13/(34.21)	15/(39.47)	0/(0)
Sperm	27/(23.68)	24/(21.05)	61/(53.51)	2/(1.75)
Total				
Vinclozolin-lineage				
Kidney	27/(21.26)	15/(11.81)	81/(63.78)	4/(3.15)
Sperm	21/(47.73)	4/(9.09)	17/(38.64)	2/(4.55)
Total	48/(28.07)	19/(11.11)	98/(57.31)	6/(3.51)

^1^Transitions

^2^Transversions

^3^Single+ Base Insertions or Deletions

^4^Double+ Base Substitutions

## Discussion

The discovery of environmentally induced germline epimutations that are transmitted transgenerationally for three or more generations following exposure of only the F1 generation fetus suggests these epimutations are not corrected by the extensive epigenetic reprogramming that normally occurs in the developing germ line [11,13]. Many environmentally induced epimutations have been reported to be manifest as abnormalities in locus- or allele-specific patterns of DNA methylation [38–42], while others appear to reflect changes in patterns of histone modifications [43]. In particular, vinclozolin has been shown to induce aberrations in DNA methylation patterns in F1 generation offspring [1,39]. Thus, studies of genome-wide DNA methylation patterns in both somatic and germ cells in descendants of pregnant dams treated with vinclozolin or vehicle only (controls) have revealed the occurrence of abundant alterations to DNA methylation patterns (epimutations) throughout the genomes of vinclozolin-lineage, but not control-lineage descendants [22]. DNA methylation epimutations were found on all chromosomes except the Y in F1-F3 generation vinclozolin-lineage descendants, but occurred most frequently in CpG-poor regions of the genome (CpG deserts) [44]. These DNA methylation epimutations were evident in F1 generation offspring of vinclozolin-treated dams and persisted until the F3 generation and beyond [39]. Specific characteristics of vinclozolin-induced transgenerational differential DNA methylation regions (DMRs) in sperm included the nonrandom occurrence of unique consensus DNA sequences including zinc finger motifs and G-quadruplex sequences [44,45]. A recent report also documented alterations in small noncoding RNAs (sncRNAs) in the sperm from F3 generation control and vinclozolin lineage rats [46].

However, inherited patterns of both DNA methylation and histone modifications are largely erased in the developing germ line and then reset so as to provide proper epigenetic programming to support development of the subsequent generation during embryonic, fetal and postnatal stages [11,47]. This germline-specific epigenetic reprogramming has the potential to correct environmentally induced epimutations, thus preventing their transmission to subsequent generations, and such a correction process has been documented in some cases [18]. Nevertheless, a growing number of cases of transgenerational transmission of environmentally induced epimutations have now been reported [2–10], indicating that exposure of individuals to environmental factors or stressful circumstances can induce epimutations that are transmitted transgenerationally and thus, somehow, escape normal germline reprogramming [19]. These germline epimutations appear to be sustained in a manner similar to imprinted genes [38], and have the potential to promote a novel etiology of various types of disease or abnormal phenotypes in the exposed individuals and in their descendants, even in the absence of any ongoing exposure to the causative agent [2,10]. Our observation that there were no significant differences in the spectra of mutations detected in F1 or F3 generation vinclozolin-lineage or control-lineage samples, respectively (Table 3), suggests that a variety of different DNA repair and/or cell death pathways were impacted by exposure to vinclozolin in a stochastic manner, thus leading to a general increase in the frequency of all types of point mutations.

The well documented alteration of DNA methylation patterns in cells of offspring exposed to vinclozolin noted above suggests that vinclozolin induces primary epimutations in the form of direct alterations of DNA methylation patterns that are subsequently propagated by epigenetic transgenerational inheritance. We tested the hypothesis that in utero exposure to vinclozolin might also induce genetic mutations that could contribute to secondary epimutations by which abnormalities in epigenetic programming result from alteration of one or more genetic functions normally required to establish such programming, because secondary epimutations would be expected to contribute to transgenerational transmission of the resulting phenotype on the basis of genetic inheritance. However, our data showing no difference in mutation frequencies between F1 generation vinclozolin- and control-lineage offspring corroborate previous reports that vinclozolin is not directly mutagenic [37]. This argues against the likelihood that exposure to vinclozolin induces secondary epimutations, or any mutator effect [39], involving an initial genetic mutation that subsequently predisposes the accelerated accumulation of additional mutations.

We next investigated mutation frequencies in F3 generation vinclozolin- and control-lineage descendants of dams that were or were not exposed to vinclozolin, respectively. Surprisingly, we detected an elevated frequency of point mutations in a subset of F3 generation vinclozolin-lineage descendants that distinguished these animals from the F3 generation control-lineage animals and from the other F3 generation vinclozolin-lineage animals. Thus, our results suggest that in utero exposure to vinclozolin promotes the initial induction of primary epimutations, at least some of which have the potential to predispose an elevated accumulation of point mutations in subsequent generations, and that this could contribute to transmission of transgenerational phenotypes. We propose that this represents a third type of epimutation which we term a “tertiary epimutation” and define as an initial disruption of epigenetic programming that leads to a subsequent change in the frequency of genetic mutations.

The addition of tertiary epimutations to the list of possible scenarios by which heritable phenotypic alterations can be introduced and/or transmitted completes the options for potential combinations of genetic and epigenetic events involved in this process (Table 4). These include 1) disruption of genomic DNA sequence leading to genetic mutations transmittable by genetic inheritance, 2) disruption of epigenetic parameters leading directly to altered epigenetic programming (primary epimutations) transmittable by epigenetic inheritance, 3) disruption of genomic DNA sequence leading to subsequent aberrations in epigenetic programming (secondary epimutations) transmittable by either genetic or epigenetic inheritance, and 4) disruption of epigenetic programming leading to the subsequent accumulation of genetic mutations at an accelerated rate (tertiary epimutations) transmittable by either epigenetic or genetic inheritance.

**Table 4:** Transmission of Genetic and Epigenetic Defects.

Type of	Initial	Manifestation	Mode of
Defect	Disruption^1^	of Disruption^2^	Transmission
Genetic Mutation	Genome	Genome	Genetic
Primary Epimutation	Epigenome	Epigenome	Epigenetic
Secondary Epimutation	Genome	Genome & Epigenome	Genetic or Epigenetic
Tertiary Epimutation	Epigenome	Epigenome & Genome	Epigenetic or Genetic

^1^Site of initial disruption

^2^Site of subsequent detectable defect

Our finding that the frequency of point mutations is not increased in vinclozolin-lineage F1 generation offspring, but is increased in a subset of vinclozolin-lineage F3 descendants exemplifies the expected gradual inheritance pattern of a tertiary epimutation. These results are consistent with a recent report of an increase in copy number variations (CNV) in F3 generation, but not F1 generation descendants of vinclozolin-exposed dams [48], and corroborate previous reports suggesting that disruption of epigenetic programming can predispose multiple types of genetic aberrations [49–58].

Abnormalities in epigenetic programming could predispose genome instability through a variety of mechanisms. The most well documented example is predisposition of C-to-T transitions via deamination of methylated cytosines [31,49,50,57,59]. Thus, a change in the methylation status of a CpG dinucleotide, particularly the addition of DNA methylation where it normally does not occur, typically increases the likelihood that a C-to-T transition will change a G-C base pair into an A-T base pair. However, we did not observe a significant increase in C-to-T transitions in samples from F3 generation vinclozolin-lineage animals (Table 3), so this does not appear to be the primary source of the increased mutation frequencies we observed in these samples.

It is well established that epigenetic programming regulates chromatin structure [60,61] and that more or less condensed chromatin appears less or more susceptible, respectively, to either spontaneous or induced mutagenesis [62,63]. We have no data describing the extent of chromatin condensation associated with the *lacI* mutation-reporter transgene in kidney or sperm cells from F3 generation vinclozolin-lineage animals, so cannot comment on whether or not this source of accelerated mutagenesis may have contributed to the elevated mutation frequencies in a subset of F3 generation vinclozolin-lineage animals.

Finally, it has been shown that specific cellular states, such as those that distinguish pluripotent and differentiated cell types, or germ and somatic cell types are correlated with more or less enhanced maintenance of genetic integrity, and that this is at least partially controlled by epigenetic mechanisms that coordinate the function of different gene networks in cells that regulate pluripotency and genetic integrity [64,65]. To the extent that this type of coordinate regulation normally governs the relative level of expression of genetic integrity (DNA repair or cell death) genes, disruptions of epigenetic programming could alter maintenance of genetic integrity resulting in the subsequent accumulation of point mutations at an accelerated rate. This would only occur if regulation of a relevant gene network was impacted by the initial epigenetic disruption, which would normally occur stochastically, such that changes in mutation frequencies would be expected to occur in only a subset of descendants of animals exposed to the initial disruptor. Our observation that only a subset of F3 vinclozolin-lineage descendants displayed significant increases in mutation frequency is consistent with this notion. In addition, a multiple step process of this sort (initial disruption of the epigenome → dysregulation of a genetic integrity gene network → an increase in the rate at which spontaneous point mutations are accumulated) would likely lead to a gradual increase in mutation frequency. This is consistent with our observation of a detectable increase in mutation frequency in F3 generation, but not in F1 generation vinclozolin-lineage descendants.

## Conclusion

Taken together, our results support a novel mechanism by which initial epimutations can predispose a subsequent accelerated accumulation of genetic mutations, and such tertiary epimutations could contribute to transgenerational transmission of defective phenotypes on the basis of either epigenetic or genetic inheritance, or both. As summarized in Table 4, tertiary epimutations complete the list of possible combinations of epigenetic and genetic effects that can contribute to the initial occurrence and subsequent transgenerational transmission of heritable phenotypic alterations. It is likely that various combinations of genetic and epigenetic mechanisms of transmission are involved in the propagation of many epimutations. Therefore future studies should include comprehensive genome-wide analyses of both mechanisms.

## Supporting Information

S1 Table (PDF)
