# Supplementary material for: Tertiary Epimutations – A Novel Aspect of Epigenetic Transgenerational Inheritance Promoting Genome Instability
Source: PLoS One. 2016 Dec 19;11(12):e0168038. doi: 10.1371/journal.pone.0168038 (PMC5167269; doi:10.1371/journal.pone.0168038)
Supplement: S1 Table — (PDF) [file pone.0168038.s001.pdf]

Tertiary Epimutations – A Novel Aspect of Epigenetic Transgenerational Inheritance Promoting Genome Instability

John R. McCarrey*, Jake D. Lehle, Seetha S. Raju, Yufeng Wang,
Eric E. Nilsson, and Michael K. Skinner

Supplementary Information – Table S1

***Correspondence:** John R. McCarrey
Department of Biology
University of Texas at San Antonio
San Antonio, TX, 78249
Email: John.McCarrey@utsa.edu
Phone: 210-458-4507

Table S1. Individual Mutations

F1 Generation Samples	Position of Mutation in *lacI* Gene*	Type of Mutation
F1CLK1	92	C > T
	116	G > T
	136	T INSERT
	180	G DEL
	180	G > A
	381	G > A
	759	T DEL
	760	T DEL
F1CLK2	57	C > T
	135	A DEL
	185	C INSERT
	234	C DEL
	702	G > A
	795	C > A
F1VLK1	42	C > T
	95	G > A
	179	C > T
	180	G > A
	760	T DEL
	882	C > T
F1VLK2	131	C > T
	135	A DEL
	198	C > T
	269	G > A
	318	C > A
	701	G > C
F1CLS1	57	C > A
	329	C > T
	539	G DEL
	620-623	CTGG DEL
	917	G > A
F1CLS3	198	C > T
	607	G DEL
	760	T DEL
	888-889	CC > TT
	918	G > A
F1CLS4	57	C > T
	87	T DEL
	92	C > T
	93	G > A

	116	G > T
	129	C > A
	759	T DEL
	760	T DEL
	843	G > A
F1CLS5	91-92	CC >TT
	93	G > A
	293	C > T
	518	G > T
	857	G > T
	955	G > C
F1VLS1	179	C > T
	270	C > T
	329	C > T
	687-688	GG > AA
	760	T DEL
	803	G > T
	894	T > A
	995	C > T
	1204	C > T
F1VLS2	35	C DEL
	56	G > A
	92	C > T
	95	G > A
	610-615	GCGTCT DEL
	665	A INSERT
	760	T DEL

**F3 Generation Samples**	**Position of Mutation in *lacI* Gene***	**Type of Mutation**
F3CLK1	81	A > C
	92	C > T
	153	T > C
	173	C DEL
	436-437	GG > TT
	792	G > A
	847	C > A
	850	C DEL
F3CLK2	32	A DEL
	68	G > T
	69	G > T
	80	C > T
	120	C > G
	125	A DEL
	348	G DEL

	386	A DEL
	388	T DEL
	453-454	AT DEL
	503	A DEL
	544	G DEL
	620-623	CTGG DEL
	696	T DEL
	791	C > T
	834	C > A
	842-843	GG > AA
	865	C DEL
	968	A DEL
	1001-1002	AA DEL
F3CLK3	75	C > T
	125	A DEL
	131	C > T
	350	A DEL
	354-384	31 BP DEL
	542	G DEL
	565	C DEL
	703	T DEL
	747	C > T
	968	A DEL
F3CLK4	83	A > G
	203	A DEL
	306	G DEL
	308	G > A
	329	C DEL
	381	G > A
	430	T DEL
	488	A DEL
	613	T DEL
	703	T DEL
	791	C > T
	936	G > C
	947-948	CT DEL
	968	A DEL
F3CLK6	44	T > G
	95	G > A
	329	C > T
	331	A DEL
	452	A DEL
	564	A DEL
	838	A DEL
	868-870	ATG DEL
	1006	A DEL
F3CLK7	72	T > C
	95	G > A
	132	G DEL

	164	A DEL
	273	C > A
	327	A DEL
	329	C > T
	511-512	CT DEL
	559	G DEL
	615	T DEL
	858	A DEL
	865-866	CT DEL
	894	T > G
	968	A DEL
	1011	C > A
F3VLK1	91-92	CC > TT
	157	G DEL
	388	T DEL
	488	A DEL
	512	T DEL
	671	G > T
	759	T DEL
	904	T DEL
	968	A DEL
F3VLK2	31	G DEL
	80	C > T
	93	G > A
	153	T DEL
	186	C > A
	270	C > T
	342	A DEL
	421	G DEL
	478	C DEL
	561-567	GTCACCA DEL
	575	G > C
	592	A DEL
	678	A DEL
	720	A DEL
	881-882	CC > TT
	899	C > T
	901	G DEL
	904	T DEL
	953	C > T
	959	C > T
	968	A DEL
	1205	C >T
F3VLK3	144	A DEL
	256	G DEL
	388	T DEL
	647	C > T
	654	A DEL
F3VLK4	27	A DEL

	56	G > A
	87	T DEL
	120	C > G
	140	G > A
	179	C > T
	194	C > G
	198	C > T
	293	C > T
	369	T DEL
	388	T DEL
	406	T DEL
	441	C DEL
	471	T DEL
	488	A DEL
	522	A DEL
	795-796	CC > AA
	868-870	ATG DEL
	882	C > T
	909	G DEL
	951	C > T
	953	C > T
	968	A DEL
F3VLK5	15	T DEL
	34	G > C
	52	T DEL
	56	G > A
	76	T DEL
	81	A > G
	174	C > T
	231	C DEL
	487	G DEL
	788	A DEL
	869	T > C
	904	T DEL
	968	A DEL
F3VLK6	38	G > C
	115	C DEL
	231	C DEL
	324	T DEL
	371	C DEL
	388	T > G
	471	T DEL
	746	A DEL
	904	T DEL
F3VLK7	26	A DEL
	42	C > T
	50	G > T
	80	C > G
	84-85	CC > TT

	92	C > G
	102-122	21 BP DEL
	122-123	GC DEL
	132	G DEL
	213	T DEL
	231	C DEL
	247-248	GC DEL
	317	T > C
	491	C DEL
	581	T DEL
	720	A DEL
	803	G > A
	828	A DEL
	868-869	AT DEL
	881	C > T
	881	C DEL
	883	G > A
	968	A DEL
F3VLK8	44	T > G
	203	A DEL
	371	C DEL
	381	G > A
	512	T DEL
	517	T DEL
	575	G > C
	809	G DEL
	904	T DEL
F3VLK9	79	T DEL
	258	C > A
	452	A DEL
	488	A DEL
	791	C > T
	886	A > T
F3VLK10	144	A DEL
	338	G DEL
	467	T DEL
	735	G DEL
	757	G DEL
	759	T DEL
	904	T DEL
	938	T DEL
F3CLS1	49	C > A
	56	G > A
	86	G > T
	95	G > A
	264	A INSERT
	270	C > T
	318	C > A
	588	G > T

	759	T DEL
	760	T DEL
F3CLS2	93	G > A
	120	A INSERT
	179	C > T
	192	A DEL
	198	C > T
	221	G > T
	620 - 623	CTGG DEL
	759	T DEL
	760	T DEL
	761	G DEL
	803	G > T
F3CLS3	92	C > T
	228	C DEL
	341	G > T
	620-623	CTGG DEL
	760	T DEL
	920	C > T
	959	C > T
F3VLS1	93	G > A
	95	G > A
	329	C > T
	628	C DEL
	759	T DEL
	760	T DEL
	959	C > T
F3VLS2	72	T > C
	179	C > T
	633	A DEL
	681	G > A
	760	T DEL
	761	G DEL
	784	C DEL
	882	C > T
F3VLS4	34	G > C
	42	C > T
	329	C > T
	331	A DEL
	491	C DEL
	681	G > A
	843	G > A
F3VLS5	42	C > T
	84-85	CC > TT
	140	G > A
	174	C > T
	180	G DEL
	760	T DEL
	770	C > T

	920	C > T
F3VLS6	178-179	CC > TT
	270	C > T
	667	A > T
	667	A DEL
	760	T DEL
	882	C > T
F3VLS7	557	A INS
	692	G DEL
	708	A DEL
	760	T DEL
	888	C > T
	993	C > A

*[Based on Big Blue Transgenic Rodent Mutagenesis Assay System Instruction Manual from Stratagene, p.26]
